# Association Between Primary Care Practice Telehealth Use and Acute Care Visits for Ambulatory Care–Sensitive Conditions During COVID-19

**DOI:** 10.1001/jamanetworkopen.2022.5484

**Published:** 2022-03-31

**Authors:** Kathleen Y. Li, Sophia Ng, Ziwei Zhu, Jeffrey S. McCullough, Keith E. Kocher, Chad Ellimoottil

**Affiliations:** 1 Institute for Healthcare Policy and Innovation, University of Michigan, Ann Arbor; 2 Department of Emergency Medicine, University of Michigan, Ann Arbor; 3 Department of Emergency Medicine, Icahn School of Medicine at Mount Sinai, New York, New York; 4 Department of Urology, University of Michigan, Ann Arbor; 5 Department of Health Management and Policy, University of Michigan, Ann Arbor

## Abstract

**Importance:**

During the COVID-19 pandemic, many primary care practices adopted telehealth in place of in-person care to preserve access to care for patients with acute and chronic conditions. The extent to which this change was associated with their rates of acute care visits (ie, emergency department visits and hospitalizations) for these conditions is unknown.

**Objective:**

To examine whether a primary care practice’s level of telehealth use is associated with a change in their rate of acute care visits for ambulatory care–sensitive conditions (ACSC visits).

**Design, Setting, and Participants:**

This retrospective cohort analysis used a difference-in-differences study design to analyze insurance claims data from 4038 Michigan primary care practices from January 1, 2019, to September 30, 2020.

**Exposures:**

Low, medium, or high tertile of practice-level telehealth use based on the rate of telehealth visits from March 1 to August 31, 2020, compared with prepandemic visit volumes.

**Main Outcomes and Measures:**

Risk-adjusted ACSC visit rates before (June to September 2019) and after (June to September 2020) the start of the COVID-19 pandemic, reported as an annualized average marginal effect. The study examined overall, acute, and chronic ACSC visits separately and controlled for practice size, in-person visit volume, zip code–level attributes, and patient characteristics.

**Results:**

A total of nearly 1.5 million beneficiaries (53% female; mean [SD] age, 40 [22] years) were attributed to 4038 primary care practices. Compared with 2019 visit volumes, median telehealth use was 0.4% for the low telehealth tertile, 14.7% for the medium telehealth tertile, and 39.0% for the high telehealth tertile. The number of ACSC visits decreased in all tertiles, with adjusted rates changing from 24.3 to 14.9 per 1000 patients per year (low), 23.9 to 15.3 per 1000 patients per year (medium), and 27.5 to 20.2 per 1000 patients per year (high). In difference-in-differences analysis, high telehealth use was associated with a higher ACSC visit rate (2.10 more visits per 1000 patients per year; 95% CI, 0.22-3.97) compared with low telehealth practices; practices in the middle tertile did not differ significantly from the low tertile. No difference was found in ACSC visits across tertiles when acute and chronic ACSC visits were examined separately.

**Conclusions and Relevance:**

In this cohort study that used a difference-in-differences analysis, the association between practice-level telehealth use and ACSC visits was mixed. High telehealth use was associated with a slightly higher overall ACSC visit rate than low telehealth practices. The association of telehealth with downstream care use should be closely monitored going forward.

## Key Points

**Question:**

What is the association between a primary care practice’s degree of telehealth use and acute care visits for ambulatory care–sensitive conditions during the COVID-19 pandemic?

**Findings:**

In this cohort study with a difference-in-differences analysis of insurance claims data from 4038 primary care practices, high primary care telehealth use was associated with 2.10 more emergency department visits or hospitalizations for ambulatory care–sensitive conditions per 1000 patients per year compared with the practices with the least telehealth use.

**Meaning:**

These findings suggest that high levels of primary care practice telehealth use may result in slightly higher acute care visits for ambulatory care–sensitive conditions.

## Teaser

This cohort study evaluates the association between practice-level telehealth use and access to care for ambulatory care–sensitive conditions during the COVID-19 pandemic.

## Introduction

The COVID-19 pandemic forced a dramatic shift from in-person care to telehealth, with an overall decrease in health care use for non–COVID-19–related medical issues, particularly in harder-hit areas, during the early months of the pandemic. This change, driven by public health measures meant to control the spread of the virus as well as public fear about contracting the virus in health care settings, has led to concerns about the negative consequences of delaying necessary medical care.^1,2^ These consequences range from delays in seeking care in emergency circumstances, such as myocardial infarction and stroke, to acute exacerbations in patients with chronic medical conditions, such as diabetes, congestive heart failure, and chronic obstructive pulmonary disease, to longer-term effects of decreased immunization rates and cancer screening.^3,4^

Before the pandemic, telehealth was an increasing but infrequently used mode of care delivery limited by a variety of factors, including lack of patient and clinician familiarity and trust in the model, challenges to accessing the supporting technology, and restrictions on reimbursement and licensing. The pandemic forced and enabled many practices to switch to delivering most care remotely via telehealth. The effect of this rapid telehealth expansion on care outcomes is uncertain. On one hand, practices that use telehealth may be able to provide better access to care when in-person visits are limited, potentially reducing emergency department (ED) visits and hospitalizations for ambulatory care–sensitive conditions (ACSC visits). Often, ACSC visits are used as an indicator of primary care access and quality and are estimated to comprise 8% of ED visits and 10% of hospitalization days annually.^5,6^ Conversely, skeptics of telehealth may argue that it is not an adequate substitute for in-person visits, and practices using telehealth may cause greater downstream demand for acute care.

By the end of the public health emergency, state and federal policy makers will need to enact permanent changes to telehealth regulations to prevent telehealth from reverting to its pre–COVID-19 state. Some recent studies^7,8^ have reported telehealth trends since COVID-19, but none to our knowledge have examined the association with patient outcomes. Given uncertainties about the long-term effect of telehealth expansion on health care spending and patient outcomes, it is critical to take advantage of the natural experiment prompted by this pandemic to determine the optimal way to shape future telehealth policy. Through this study, we aimed to evaluate the association between practice-level telehealth use and access to care on an important short- to medium-term outcome: ED visits and hospitalizations for ACSCs. The findings reported here contain a more detailed analysis over a longer measurement period of telemedicine use (March 1 to August 31, 2020, compared with March 1 to July 31, 2020) of findings published in an earlier policy brief.^9^

## Methods

### Design and Participants

In this cohort study, we retrospectively analyzed insurance claims and member enrollment data from a large commercial insurer from January 1, 2019, through September 30, 2020, using a difference-in-differences design. Our study population included beneficiaries enrolled continuously throughout this period and who had at least 1 outpatient evaluation and management visit that could be used to attribute them to a primary care practice using the Centers for Medicare & Medicaid Services’ 2-step attribution method based on each practice’s taxpayer identification number.^10^ Practices with fewer than 10 attributed beneficiaries or fewer than 10 claims during the period from January 1, 2019, to February 29, 2020, were excluded from analysis. Practices that were extreme outliers (>99th percentile) in their telehealth use, defined below, were also excluded. This study was determined to be exempt from review and the requirement for informed consent by the University of Michigan Institutional Review Board because the data used in the study had been deidentified and coded. This study followed the Strengthening the Reporting of Observational Studies in Epidemiology (STROBE) reporting guideline.

### Defining Practice-Level Telehealth Use

To compare practices with different volumes of claims, we defined a practice’s telehealth use relative to the pre–COVID-19 period as the number of telehealth visits from March 1 to August 31, 2020, divided by the number of monthly in-person and telehealth visits from January 1, 2019, to February 29, 2020. We used a period of 14 months in the denominator to obtain a more stable monthly estimate. Similarly, because a degree of in-person visits continued throughout the pandemic, we also defined an analogous term to measure the proportion of in-person visits that continued compared with the pre–COVID-19 period as the number of monthly in-person visits from March 1 to August 31, 2020, divided by the number of monthly in-person and telehealth visits from January 1, 2019, to February 29, 2020. The sum of telehealth and in-person use reflects the percentage of care provided by a given practice compared with its own prepandemic volume. We stratified practices into low, medium, and high tertiles of telehealth use as our exposure variable.

### Practice and Patient Characteristics

We adjusted for a number of patient- and practice-level characteristics that may affect telehealth use and ACSC visits. The decision to adopt telehealth likely depended on a number of practice-specific factors: clinician motivation and comfort with technology, perceived patient demand, cost of infrastructure investment, and anticipated reimbursement. We examined practice size (count of national provider identifiers associated with a given practice’s tax identification number) and geographic characteristics associated with a practice’s zip code, including rural or urban status and percentage of households with broadband access. Beneficiaries attributed to practices that spanned multiple zip codes were associated with the zip code characteristics for which that individual had the most claims during the study period.

A practice’s case mix will affect how often its patients may need acute care, particularly for ACSCs. A family medicine practice with primarily healthy younger patients likely has fewer patients with ACSC visits than a practice with many older patients with multiple comorbidities. We performed risk adjustment using patients’ age, sex, and presence of comorbid conditions within the prior year, including congestive heart failure, cancer, kidney insufficiency, chronic obstructive pulmonary disease, diabetes, and immune compromise. These condition categories were identified through *International Statistical Classification of Diseases and Related Health Problems, Tenth Revision* (*ICD-10*) diagnoses using the Centers for Medicare & Medicaid Services Hierarchical Condition Categories 2016 risk adjustment model.^11^

### Outcome Measures

Our primary outcome was the difference in the risk-adjusted rate of ACSC visits from before the start of the pandemic (June 1 to September 30, 2019) and after (June 1 to September 30, 2020) for high- and medium-tertile primary care practices compared with practices in the lowest tertile of telehealth use, estimated using average marginal effects (AMEs). We excluded March 1 to May 31, 2020, from measurement because of sudden and rapid pandemic-related fluctuations in all forms of care (outpatient, ED, and hospitalizations). Owing to the low frequency of ACSC visits at a population level (<1% of beneficiaries experienced an ACSC visit), we considered ACSC visits to be a binary outcome. We measured ACSC visits in aggregate as well as separated into acute and chronic ACSC composite measures as defined by the Centers for Medicare & Medicaid Services using *ICD-10* codes.^12^ We also performed a subgroup analysis that included only beneficiaries with preexisting diabetes, congestive heart failure, chronic obstructive pulmonary disease, or kidney insufficiency.

### Statistical Analysis

We used the χ^2^ and Kruskal-Wallis tests to examine differences in telehealth adoption and use rates across practice characteristics. We performed univariate and multivariable logistic regression analysis by using a difference-in-differences model to test the association between the tertile of telehealth use (referred to as low, medium, or high telehealth use) and ACSC visit rates, using the lowest tertile as the reference group and accounting for clustering at the practice level. This analysis tests whether there were differences in our study outcomes among exposed (medium or high telehealth use) practices compared with control (low telehealth use) practices before and after the pandemic started (June 1 to September 31, 2019, vs June 1 to September 31, 2020). The difference-in-differences design accounts for decreases in acute care visits related to external factors, such as public health guidance.^13,14^ We then used AMEs to estimate the association of practice telehealth use during the pandemic with the rate of ACSC visits. Because our prepandemic and postpandemic measurement periods only span 4 months, these associations were annualized to report the estimated rate of ACSC visits per 1000 patients per year.^15^ Additional sensitivity analyses were performed using binary (none vs any telehealth adoption) and continuous rates of telehealth use as opposed to tertiles to assess the robustness of our findings. We conducted 2-sided hypothesis tests with a significance level of α = .05. All analyses were performed in Stata statistical software, version 16 (StataCorp LLC).

## Results

### Population Characteristics

Our study population included approximately 1.8 million individuals with continuous enrollment during the study period and who could be attributed to a primary care practice. Initially, 6099 primary care practices were identified. After excluding practices with fewer than 10 beneficiaries or 10 prepandemic claims (n = 1980) and practices with greater than the 99th percentile telemedicine use (n = 41), nearly 1.5 million individuals (53% female; mean [SD] age, 40 [22] years) attributed to 4038 primary care practices were included for analysis. The characteristics of the study participants are given in Table 1. Race and ethnicity data were not available in our data set. Of practices that met the inclusion criteria, 47% were solo practices, 36% were small (2-5 clinicians), 13% were medium (6-20 clinicians), and 4% were large (≥21 clinicians). Solo practices made up 11% of outpatient claims, whereas large practices made up 59% of claims.

**Table 1.** Demographic Characteristics and Acute Care Use of Blue Cross Blue Shield of Michigan Beneficiaries by Primary Care Attribution Status^a^

Characteristic	Attributed to PCP (n = 1 490 734)	No PCP (n = 32 436)
Age, mean (SD), y	39.8 (22.2)	31.4 (15.1)
Sex		
Female	52.9	38.6
Male	47.1	61.4
Comorbidities^b^		
Cancer	4.7	0.2
Diabetes	10.0	0.8
Immune compromise	1.4	0.2
CHF	3.1	0.2
COPD	3.7	0.4
CKD	2.1	0.4
Telehealth visits (March to August 2020)^c^	0.5	0.0
Acute care visits (January 2019 to September 2020)^c^		
Emergency department	0.49	0.23
Hospitalizations	0.09	0.03
ACSC visits		
Any^d^	0.04	0.02
Acute	0.03	0.01
Chronic	0.02	0.01

Abbreviations: ACSC, ambulatory care–sensitive condition; CHF, congestive heart failure; CKD, chronic kidney disease; COPD, chronic obstructive pulmonary disease; PCP, primary care practice.

^a^ Data are presented as percentages unless otherwise indicated. Data are from an analysis of Blue Cross Blue Shield of Michigan claims for 2019 to 2020.

^b^ Comorbidities identified from hierarchical condition categories.

^c^ Mean visits per beneficiary.

^d^ Emergency department visits or hospitalizations for ACSCs identified using *International Statistical Classification of Diseases and Related Health Problems, Tenth Revision* (*ICD-10*) codes.

### Changes in Visit Volume

Before the pandemic, the mean (SD) number of primary care practice visits each month was 824 768 (43 250). Compared with prepandemic visit volumes, there were 27% fewer overall visits from March 1 to August 31, 2020. In-person visit rates decreased by 42% compared with 2019 visits, and telehealth visits in the post–COVID-19 period comprised 19% of 2019 visit volumes. More than 1 in 4 practices (29%) had no telehealth claims identified—these practices tended to be smaller and more likely to be in rural areas. Compared with 2019 visit volumes, median practice telehealth use was 0.4% (IQR, 0.0%-3.8%) for the low tertile, 14.7% (IQR, 12.7%-17.3%) for the medium tertile, and 39.0% (IQR, 28.0%-59.8%) for the high tertile. Patient and practice characteristics for each tertile are listed in Table 2. Practices in the high telehealth tertile were generally larger (number of patients per practice, 148 [IQR, 44-441] in the high tertile vs 66 [IQR, 25-225.5] in the low tertile and 206 [IQR, 50-588.5] in the medium tertile), were less rural (rural practices, 8.2% vs 24.2% in the low tertile and 18.1% in the medium tertile), had patients who were older (median [IQR] age, 46 [27-59] years vs 34 [15-54] years in the low tertile and 43 [21-58] years in the medium tertile), and had more comorbidities (diabetes, 12.2% vs 7.8% in the low tertile and 10.0% in the medium tertile) than low telehealth practices.

**Table 2.** Differences in Low–, Medium–, and High–Telehealth Use Primary Care Practices^a^

Characteristic	Tertile of telehealth use		
	Low (495 665 beneficiaries and 2532 PCPs)	Medium (500 815 beneficiaries and 512 PCPs)	High (488 967 beneficiaries and 995 PCPs)
Practice, median (IQR), %^b^			
Telehealth use	0.4 (0.0-3.8)	14.7 (12.7-17.3)	39.0 (28.0-59.8)
Practice in-person use	62.6 (47.6-74.8)	60.3 (49.0-71.1)	43.3 (27.5-57.9)
Overall visit rate vs 2019, median (IQR), %	65.2 (49.2-77.5)	74.9 (64.4-85.6)	86.7 (74.6-103.3)
Per practice, median (IQR), No.			
Clinicians	2 (1-3)	2 (1-5)	2 (1-4)
Patients	65 (25-225.5)	206 (50-588.5)	148 (46-441)
Rural, weighted % of practices^c^	24.2	18.1	8.2
Sex, %			
Female	52.2	53.8	52.7
Male	47.8	46.2	47.2
Age, median (IQR), y	34 (15-54)	43 (21-58)	46 (27-59)
Rural, % of beneficiaries	26.3	22.0	10.7
Comorbidities, % of beneficiaries^d^			
Cancer	3.3	5.2	5.9
Diabetes	7.8	10.0	12.2
Immune compromise	0.9	1.5	1.8
CHF	2.4	3.0	3.8
COPD	3.1	3.7	4.5
CKD	1.4	2.0	2.8

Abbreviations: CHF, congestive heart failure; CKD, chronic kidney disease; COPD, chronic obstructive pulmonary disease; PCP, primary care practice.

^a^ Data are from an analysis of Blue Cross Blue Shield of Michigan claims for 2019 to 2020.

^b^ Percentage of visits compared with mean monthly visits of any modality (January 2019 to February 2020).

^c^ Rural was defined using the practice’s zip code(s) as classified in the Federal Office of Rural Health Policy Data Files. Frequency weights are based on the number of beneficiaries attributed to each practice–zip code combination.

^d^ Comorbidities identified from hierarchical condition categories.

### Changes in ACSC Visits

**Figure 1.**
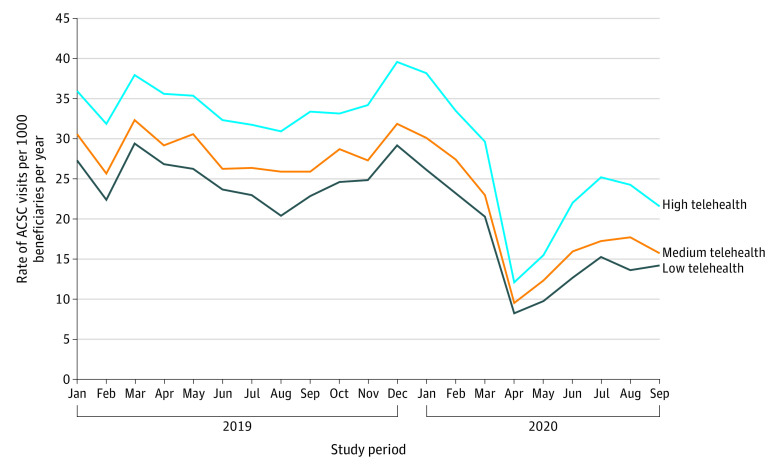
Unadjusted Rate of Aggregate Ambulatory Care–Sensitive Condition (ACSC) Visits Over Time by Level of Practice Telehealth Use Data are based on an analysis of Blue Cross Blue Shield of Michigan claims for 2019 to 2020. Low, medium, and high telehealth tertiles are derived from primary care practice telehealth use from March to August 2020 compared with 2019 visit volume, weighted by number of patients per practice.

Visits for ACSCs comprised 8% of all ED visits and hospitalizations. Rates of beneficiaries with ACSC visits followed a similar pattern for all tertiles of telehealth use during the study period, with a sharp decrease in visits during March 1 to April 30, 2020, followed by a partial rebound in the subsequent months (Figure 1). Overall, there were 8.4 (25.3 annualized) ACSC visits per 1000 patients in the pretelehealth period of June 1 to September 31, 2019, and 5.6 (16.9 annualized) in the posttelehealth period of June 1 to September 31, 2020.

### Difference in ACSC Visits by Practice Telehealth Tertile

**Figure 2.**
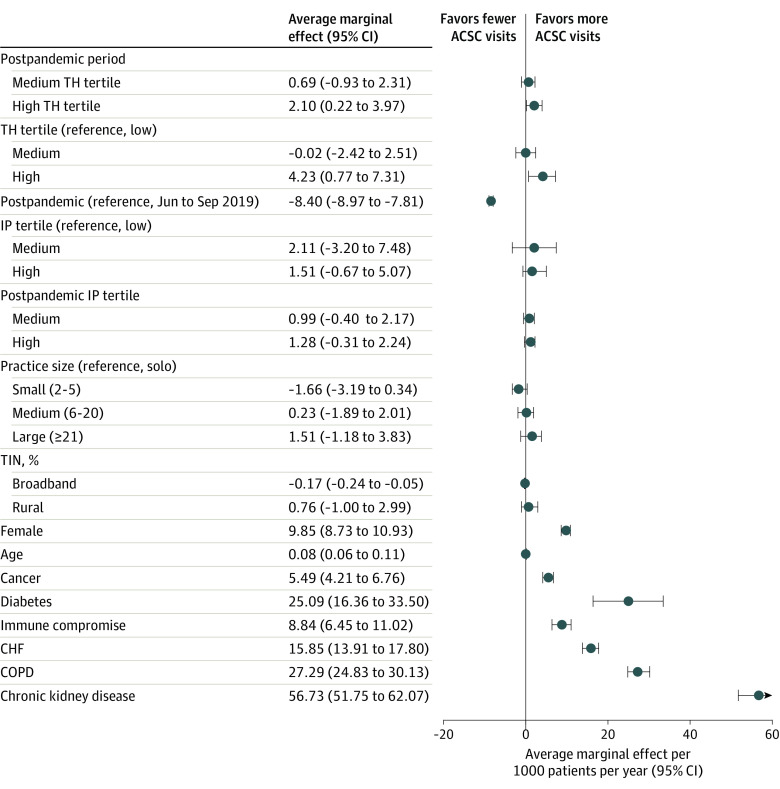
Average Marginal Effect of Practice Telehealth (TH) Use, Practice Characteristics, and Patient Characteristics on Ambulatory Care–Sensitive Condition (ACSC) Visits per 1000 Patients per Year Data are based on an analysis of Blue Cross Blue Shield of Michigan claims for 2019 to 2020. The primary outcome is the average marginal effect of the interaction between TH tertile (low, medium, or high) and the study period. The average marginal effects of the other covariates are shown for comparison. Error bars indicate 95% CIs. CHF indicates congestive heart failure; COPD, chronic obstructive pulmonary disease; IP, in-person; and TIN, taxpayer identification number.

In unadjusted difference-in-differences analysis, practices with medium or high levels of telehealth use did not have statistically different ACSC visit rates compared with those with the lowest level of telehealth use (AME, −0.86 visits per 1000 patients per year; 95% CI, −2.49 to 0.76 for the medium tertile; AME, −0.30; 95% CI, −2.03 to 1.43 for the high tertile). After adjustment for individual patient- and practice-level covariates and accounting for pre–COVID-19 differences in ACSC visit rates, high practice telehealth use was associated with slightly higher ACSC visit rates than low telehealth practices (AME, 2.10; 95% CI, 0.22-3.97), but those in the medium telehealth tertile did not differ significantly (Table 3). Of note, factors such as kidney insufficiency (AME, 56.73; 95% CI, 51.75-62.07), chronic obstructive pulmonary disease (AME, 27.29; 95% CI, 24.83-30.13), diabetes (AME, 25.09; 95% CI, 16.36-33.50), and congestive heart failure (AME, 15.85; 95% CI, 13.91-17.80) had a much greater association with estimated ACSC visit rates than telehealth use (Figure 2). When modeling acute and chronic ACSCs separately, we found no association between higher telehealth use and ACSC visit rates (Table 3). Full regression results are available in eTable 1 in the Supplement. In the subgroup analysis, among beneficiaries with at least 1 chronic condition linked to an ACSC (diabetes, congestive heart failure, chronic obstructive pulmonary disease, or kidney insufficiency in 215 105 beneficiaries across 3293 practices), practices with high compared with low telehealth use did not have statistically different ACSC visit rates (AME, 6.76; 95% CI, −3.4 to 16.95).

**Table 3.** Difference-in-Differences Estimate for the Association of High Telemedicine Adoption With Rates of Acute, Chronic, and Aggregate ACSC Visits per 1000 Patients per Year^a^

Tertile of telehealth use	Visits per 1000 patients per year			Difference in differences (95% CI)^b^
	Prepandemic period (June to September 2019)	Postpandemic period (June to September 2020)	Difference	
All ACSC visits^c^				
Low (reference)	24.30	14.93	−9.37	NA
Medium	23.94	15.26	−8.68	0.69 (−0.93 to 2.31)
High	27.48	20.21	−7.28	2.10 (0.22 to 3.97)
Acute ACSC composite				
Low (reference)	16.28	10.48	−5.79	NA
Medium	15.91	10.63	−5.27	0.52 (−0.85 to 1.89)
High	17.90	12.98	−4.92	0.87 (−0.50 to 2.24)
Chronic ACSC composite				
Low (reference)	8.57	4.67	−3.90	NA
Medium	8.55	4.86	−3.69	0.21 (−0.64 to 1.07)
High	10.14	7.44	−2.69	1.21 (−0.22 to 2.64)

Abbreviations: ACSC, ambulatory care–sensitive condition; NA, not applicable.

^a^ Data are from an analysis of Blue Cross Blue Shield of Michigan claims for 2019 to 2020.

^b^ Estimated using average marginal effects.

^c^ Ambulatory care–sensitive condition visits per 1000 patients per year estimated using estimated margins.

### Sensitivity Analysis

In a sensitivity analysis that used a binary definition of telehealth use, any telehealth adoption was also associated with a higher overall ACSC visit rate (AME, 3.32; 95% CI, 0.17-6.46) and higher chronic ACSC visit rate (AME, 2.18; 95% CI, 0.10-4.27), but no association was found with acute ACSC visits (AME, 1.14; 95% CI, −1.10 to 3.37) (eTable 2 in the Supplement). When we examined telehealth use as a continuous variable, an increase in telehealth use from 0% to 100% relative to 2019 visits, taking into account prepandemic differences, was not associated with different ACSC visit rates both overall (AME, 1.02; 95% CI, −1.73 to 3.77) and when considering separately the acute (AME, 0.42; 95% CI, −1.73 to 2.57) and chronic (AME, 0.38; 95% CI, −1.30 to 2.06) ACSC visit composites (eTable 3 in the Supplement).

## Discussion

In this cohort study using a difference-in-differences analysis, we found that high practice telehealth use was associated with a small increase in ACSC visits compared with low practice telehealth use. However, ACSC visit rates were not significantly different between medium and low telehealth practices. When acute and chronic ACSCs were evaluated separately, we found no association between telehealth use and ACSC visits.

Overall primary care telehealth use in our study parallels trends reported in other studies^16,17^ examining telehealth use during this time. Patel et al^7^ found that, from March through June 2020, telemedicine made up approximately 30% of all visits, yet total outpatient visits decreased by 35% compared with the pre–COVID-19 period.

Our finding that beneficiaries of practices that used more telehealth had slightly higher rates of ACSC visits was unexpected based on prior literature,^18,19,20^ although existing studies^18,19^ examined telehealth use in a narrower context. A study by Jia et al^18^ found decreased hospitalizations for ACSCs among veterans with diabetes given home telehealth access, although this telehealth technology did not involve audio-video communication. Our study examined the association between primary care practice–level telehealth adoption on ACSC visits among a more general population but within a shorter time frame. A 2014 systematic review by Bashshur et al^19^ on telemedicine in chronic disease management found that studies of video teleconferencing reported neutral to decreased hospitalizations and length of stay for patients with congestive heart failure and fewer or no associations with readmissions for chronic obstructive pulmonary disease. A more recent qualitative study^20^ of primary care physician perspectives on telemedicine found it to be adequate for visits for reviewing laboratory test results, medication reconciliation, and chronic disease management but less well suited for examining specific patient concerns, such as chest pain, abdominal pain, neurologic symptoms, and rashes.

There are several explanations for our findings. The fact that telehealth did not reduce ACSC visits during a period when overall health care use was down may be more a reflection of deferred or reduced access to routine care during the pandemic than an indication of telehealth quality itself. The association between more practice-level telehealth use and higher rates of ACSCs may be confounded by patient acuity. Some patients may have put off seeing a physician—even virtually—until their condition necessitated an ED visit or hospitalization. Practices with higher rates of telehealth use had a higher baseline visit rate for ACSCs than those with lower rates of telehealth use, suggesting that their patients may have more severe or more complex illness in ways not captured in claims data. On the other hand, the fact that telehealth was not associated with even greater increases in ACSC visits suggests that it may be an adequate substitute for in-person care in many situations. How widespread telehealth use may affect ACSC visits in the long term, outside a pandemic setting with fluctuations in multiple aspects of care use, remains to be seen.

### Limitations

Our study has a number of limitations. Administrative claims data do not include clinical information; thus, we cannot fully differentiate the severity of illness during outpatient and ED visits before and during the initial COVID-19 surge, although we adjusted for several individual patient covariates. Our data set also did not include data on race and ethnicity, therefore limiting our ability to examine potential differences in ACSC visit trends among these groups. Ambulatory care–sensitive conditions are a measure of population-level access to high-quality primary care; therefore, we were interested in a practice’s telehealth use as a proxy for an individual’s access to primary care. Our data also included only claims and enrollment information for a single commercial payer within Michigan, so telehealth adoption and ACSC visit rates may not reflect a practice’s overall performance, particularly if their payer mix is more heavily weighted to other insurers, including Medicare or Medicaid. In addition, our analysis was limited to those beneficiaries with continuous enrollment throughout the study period. The same populations who experience disruptions in health insurance coverage may also have difficulty accessing both telehealth and in-person services as well as different patterns of acute care use.^21,22^ Examining how telehealth may alleviate or exacerbate disparities in care is an important area of research outside the scope of our study. Finally, our study examined primary care access in the first 6 months of the pandemic and ACSC visits in the first 7 months, whereas ACSC visits are typically measured and evaluated on a longer time scale. Additional research is needed to more accurately determine the long-term effect of expanded primary care telehealth on ACSC visits.

## Conclusions

As telehealth continues to mature as a mode of health care delivery, it will be important to evaluate not only telehealth access and adoption but also quality and value in the context of overall health care use. Identifying the optimal use of telehealth services by maximizing needed access while minimizing low-value care will benefit patients, health care systems, and private and public payers alike. This cohort study looks at not only variation in telehealth use but also the association between the degree of telehealth use and ACSC visits, a widely used measure of primary care access and quality. Although future research is needed to examine other aspects of telehealth care quality and best practices, our findings suggest that high rates of telehealth use early in the pandemic were associated with, at most, minimal increases in primary care–sensitive acute care visits.

## Notes

Supplement. **eTable 1.** Multivariable Logistic Regression Results of Association Between Telehealth Use Tertile and ACSC Visits **eTable 2.** Average Marginal Effects of Practice Telehealth Use (Binary) and Practice- And Patient-Level Characteristics on Estimated ACSC Visits per 1000 Patients per Year **eTable 3.** Average Marginal Effects of Practice Telehealth Use (Continuous) and Practice- and Patient-Level Characteristics on Estimated ACSC Visits per 1000 Patients per Year

## References

[1] Gonzalez D, Karpman M, Kenney GM, Zuckerman S. *Delayed and Forgone Health Care for Nonelderly Adults During the COVID-19 Pandemic*. Urban Institute; 2021. Accessed July 29, 2021. https://www.urban.org/sites/default/files/publication/103651/delayed-and-forgone-health-care-for-nonelderly-adults-during-the-covid-19-pandemic_1.pdf

[2] Chen J, McGeorge R. Spillover effects of the COVID-19 pandemic could drive long-term health consequences for non-COVID-19 patients. *Health Affairs *blog. Posted October 23, 2020. Accessed July 29, 2021. https://www.healthaffairs.org/do/10.1377/hblog20201020.566558/full/

[3] Moser DK, Kimble LP, Alberts MJ, ; American Heart Association Council on Cardiovascular Nursing and Stroke Council. Reducing delay in seeking treatment by patients with acute coronary syndrome and stroke: a scientific statement from the American Heart Association Council on Cardiovascular Nursing and Stroke Council. J Cardiovasc Nurs. 2007;22(4):326-343. doi:10.1097/01.JCN.0000278963.28619.4a 17589286

[4] Bhuyan N. Beware consequences of delaying primary care in pandemic. June 25, 2020. Accessed September 8, 2021. https://www.aafp.org/news/blogs/freshperspectives/entry/20200625fp-coviddelay.html

[5] Johnson PJ, Ghildayal N, Ward AC, Westgard BC, Boland LL, Hokanson JS. Disparities in potentially avoidable emergency department (ED) care: ED visits for ambulatory care sensitive conditions. Med Care. 2012;50(12):1020-1028. doi:10.1097/MLR.0b013e318270bad4 23032354

[6] Australian Institute of Health and Welfare. Potentially preventable hospitalisations in Australia by age groups and small geographic areas, 2017–18. Accessed June 21, 2021. https://www.aihw.gov.au/reports/primary-health-care/potentially-preventable-hospitalisations/contents/overview

[7] Patel SY, Mehrotra A, Huskamp HA, Uscher-Pines L, Ganguli I, Barnett ML. Variation in telemedicine use and outpatient care during the COVID-19 pandemic in the United States. Health Aff (Millwood). 2021;40(2):349-358. doi:10.1377/hlthaff.2020.01786 33523745 PMC7967498

[8] Schweiberger K, Hoberman A, Iagnemma J, . Practice-level variation in telemedicine use in a pediatric primary care network during the COVID-19 pandemic: retrospective analysis and survey study. J Med internet Res. 2020;22(12):e24345. doi:10.2196/24345 33290244 PMC7752181

[9] Li KY, Ng S, McCullough J, Keith ZZK, Ellimootil C. Telehealth Use in Michigan During COVID-19: Variation in Primary Care Telehealth Adoption and Its Impact on Emergency Department Use and Hospitalizations. Institute for Healthcare Policy and Innovation, University of Michigan; 2021.

[10] *Fact Sheet: Two-Step Attribution for Claims-Based Quality Outcome Measures and per Capita Cost Measures Included in the Value Modifier*. Centers for Medicare & Medicaid Services; 2017. Accessed June 17, 2021. https://www.cms.gov/Medicare/Medicare-Fee-for-Service-Payment/PhysicianFeedbackProgram/Downloads/2016-Attribution-Fact-Sheet.pdf

[11] Centers for Medicare & Medicaid Services. Risk adjustment. Accessed March 15, 2021. https://www.cms.gov/Medicare/Health-Plans/MedicareAdvtgSpecRateStats/Risk-Adjustors

[12] *2016 Measure Information About the Hospital Admissions for Acute and Chronic Ambulatory Care-Sensitive Condition (ACSC) Composite Measures, Calculated for the 2018 Value-Based Payment Modifier Program*. Centers for Medicare & Medicaid Services; 2017. Accessed June 8, 2021. https://www.cms.gov/Medicare/Medicare-Fee-for-Service-Payment/PhysicianFeedbackProgram/Downloads/2016-ACSC-MIF.pdf

[13] Hartnett KP, Kite-Powell A, DeVies J, ; National Syndromic Surveillance Program Community of Practice. Impact of the COVID-19 pandemic on emergency department visits—United States, January 1, 2019-May 30, 2020. MMWR Morb Mortal Wkly Rep. 2020;69(23):699-704. doi:10.15585/mmwr.mm6923e1 32525856 PMC7315789

[14] Smulowitz PB, O’Malley AJ, Khidir H, Zaborski L, McWilliams JM, Landon BE. National trends in ED visits, hospital admissions, and mortality for Medicare patients during the COVID-19 pandemic. Health Aff (Millwood). 2021;40(9):1457-1464. doi:10.1377/hlthaff.2021.00561 34495730

[15] Williams R. Using the margins command to estimate and interpret adjusted predictions and marginal effects. Stata. 2012;12(2):308-331. doi:10.1177/1536867X1201200209

[16] Alexander GC, Tajanlangit M, Heyward J, Mansour O, Qato DM, Stafford RS. Use and content of primary care office-based vs telemedicine care visits during the COVID-19 pandemic in the US. JAMA Netw Open. 2020;3(10):e2021476. doi:10.1001/jamanetworkopen.2020.21476 33006622 PMC7532385

[17] Mafi JN, Craff M, Vangala S, . Trends in US ambulatory care patterns during the COVID-19 pandemic, 2019-2021. JAMA. 2022;327(3):237-247. doi:10.1001/jama.2021.24294 35040886 PMC8767442

[18] Jia H, Chuang HC, Wu SS, Wang X, Chumbler NR. Long-term effect of home telehealth services on preventable hospitalization use. J Rehabil Res Dev. 2009;46(5):557-566. doi:10.1682/JRRD.2008.09.0133 19882490

[19] Bashshur RL, Shannon GW, Smith BR, . The empirical foundations of telemedicine interventions for chronic disease management. Telemed J E Health. 2014;20(9):769-800. doi:10.1089/tmj.2014.9981 24968105 PMC4148063

[20] Gomez T, Anaya YB, Shih KJ, Tarn DM. A qualitative study of primary care physicians’ experiences with telemedicine during COVID-19. J Am Board Fam Med. 2021;34(suppl):S61-S70. doi:10.3122/jabfm.2021.S1.200517 33622820

[21] Park J, Erikson C, Han X, Iyer P. Are state telehealth policies associated with the use of telehealth services among underserved populations? Health Aff (Millwood). 2018;37(12):2060-2068. doi:10.1377/hlthaff.2018.05101 30633679

[22] Zhang D, Shi L, Han X, . Disparities in telehealth utilization during the COVID-19 pandemic: findings from a nationally representative survey in the United States. J Telemed Telecare. 2021;X211051677. Published online October 11, 2021. doi:10.1177/1357633X211051677 34633882

