# Supplementary material for: Association Between Primary Care Practice Telehealth Use and Acute Care Visits for Ambulatory Care–Sensitive Conditions During COVID-19
Source: JAMA Netw Open. 2022 Mar 31;5(3):e225484. doi: 10.1001/jamanetworkopen.2022.5484 (PMC8972029; doi:10.1001/jamanetworkopen.2022.5484)
Supplement: Supplement. — eTable 1. Multivariable Logistic Regression Results of Association Between Telehealth Use Tertile and ACSC Visits eTable 2. Average Marginal Effects of Practice Telehealth Use (Binary) and Practice- And Patient-Level Characteristics on Estimated ACSC Visits per 1000 Patients per Year eTable 3. Average Marginal Effects of Practice Telehealth Use (Continuous) and Practice- and Patient-Level Characteristics on Estimated ACSC Visits per 1000 Patients per Year [file jamanetwopen-e225484-s001.pdf]

Supplementary Online Content

Li KY, Ng S, Zhu Z, McCullough JS, Kocher KE, Ellimoottil C. Association between primary care practice telehealth use and acute care visits for ambulatory care–sensitive conditions during COVID-19. *JAMA Netw Open*. 2022;5(3):e225484. doi:10.1001/jamanetworkopen.2022.5484

eTable 1. Multivariable Logistic Regression Results of Association Between Telehealth Use Tertile and ACSC Visits

eTable 2. Average Marginal Effects of Practice Telehealth Use (Binary) and Practice- And Patient-Level Characteristics on Estimated ACSC Visits per 1000 Patients per Year

eTable 3. Average Marginal Effects of Practice Telehealth Use (Continuous) and Practice- and Patient-Level Characteristics on Estimated ACSC Visits per 1000 Patients per Year

This supplementary material has been provided by the authors to give readers additional information about their work.

© 2022 Li KY et al. *JAMA Network Open.*

eTable 1 Multivariable logistic regression results of association between telehealth use tertile and ACSC visits

Attribute	Any ACSC[a] Odds Ratio (95% CI)	p	Acute ACSC Odds Ratio (95%CI)	p	Chronic ACSC Odds Ratio (95% CI)	p
**TH tertile # period[b]**						
Medium - post	1.04 (0.94-1.15)	0.45	1.04 (0.93-1.16)	0.50	1.04 (0.88-1.23)	0.61
High - post	1.20 (1.08-1.34)	0.001	1.13 (1.02-1.25)	0.02	1.35 (1.10-1.66)	0.004
**TH tertile**						
Medium	0.98 (0.88-1.10)	0.79	0.98 (0.89-1.08)	0.64	1.00 (0.83-1.19)	0.98
High	1.14 (1.01-1.28)	0.04	1.10 (0.98-1.24)	0.10	1.19 (1.03-1.37)	0.02
**In-person tertile[c]**						
Medium	1.07 (0.87-1.31)	0.52	1.07 (0.92-1.25)	0.37	1.07 (0.80-1.42)	0.67
High	1.04 (0.94-1.14)	0.45	1.04 (0.95-1.13)	0.39	1.03 (0.90-1.19)	0.64
**Period (ref=Jun-Sep 2019)**						
Post (Jun-Sep 2020)	0.57 (0.51-0.63)	0.000	0.61 (0.55-0.68)	0.000	0.49 (0.42-0.58)	0.000
**IP tertile # period**						
Medium - post	1.10 (0.95-1.27)	0.20	1.06 (0.98-1.14)	0.12	1.16 (0.82-1.64)	0.40
High - post	1.10 (1.00-1.21	0.04	1.08 (0.98-1.21)	0.13	1.12 (0.96-1.32)	0.15
**Practice size category[d] (ref = solo)**						
Small (2-5)	0.92 (0.85-0.99)	0.03	0.97 (0.89-1.05)	0.40	0.84 (0.73-0.96)	0.009
Medium (6-20)	1.01 (0.91-1.13)	0.83	1.09 (0.98-1.21)	0.10	0.86. (0.73-1.02)	0.08
Large (21+)	1.08 (0.95-1.22)	0.26	1.07 (0.97-1.17)	0.17	1.10 (0.90-1.36)	0.36
TIN percent with broadband	0.99 (0.99-1.00)	0.000	0.99 (0.99-1.00)	0.000	0.99 (0.98-1.00)	0.001
TIN rural	1.04 (0.95-1.13)	0.40	1.11 (1.04-1.20)	0.003	0.90 (0.79-1.04)	0.16
Female	1.64 (1.59-1.69)	0.000	2.02 (1.86-2.18)	0.000	1.11 (1.04-1.18)	0.001
Age	1.00 (1.00-1.00)	0.000	1.01 (1.01-1.01)	0.000	0.99 (0.99-1.00)	0.000
**Comorbidities[e]**						
Cancer	1.28 (1.21-1.35)	0.000	1.51 (1.42-1.60)	0.000	0.94 (0.85-1.03)	0.19
Diabetes	2.55 (2.08-3.13)	0.000	1.33 (1.26-1.40)	0.000	7.33 (4.75-11.31)	0.000
Immune compromise	1.45 (1.33-1.57)	0.000	1.57 (1.45-1.70)	0.000	1.16 (1.00-1.35)	0.05
CHF	1.86 (1.73-1.99)	0.000	1.55 (1.44-1.66)	0.000	2.41 (2.08-2.79)	0.000
COPD	2.54 (2.33-2.77)	0.000	1.75 (1.65-1.86)	0.000	4.23 (3.40-5.27)	0.000
Renal insufficiency	4.37 (3.87-4.93)	0.000	6.45 (5.86-7.10)	0.000	2.26. (2.00-2.55)	0.000

Source: Analysis of Blue Cross Blue Shield of Michigan claims, 2019-2020
[a]ACSC: Emergency department visits or hospitalizations for ambulatory care-sensitive conditions
[b]Interaction between telehealth tertile (low, medium, high) and the time period (pre vs post)
[c]Proportion of care provided in-person in March-Aug 2020 compared to total visits January 2019-February 2020
[d]Practice size determined by number of National Provider Identifier (NPI) numbers associated with the same Tax Identification Number (TIN)
[e]Comorbidities identified from Hierarchical Condition Categories

© 2022 Li KY et al. *JAMA Network Open.*

eTable 2 Average marginal effects of practice telehealth use (binary) and practice- and patient-level characteristics on estimated ACSC visits per 1000 patients per year

Attribute	Any ACSC[a] AME (95% CI)	Acute ACSCs AME (95% CI)	Chronic ACSCs AME (95% CI)
**Any telehealth use # period[b]**			
Yes - post	3.32 (0.17 - 6.46)	1.14 (-1.10 - 3.37)	2.18 (0.10 - 4.27)
**Any telehealth use (ref = no)**	0.99 (-0.72 - 2.7)	0.99 (-0.29 - 2.27)	0.20 (-0.88 - 1.27)
**Period (ref=Jun-Sep 2019)**			
Post (Jun-Sep 2020)	-8.42 (-9.10 - -7.74)	-5.33 (-5.77 - -4.89)	-3.39 (-3.95 - -2.83)
**In-person visit rate[c]**	-0.01 (-1.25 - 1.23)	0.06 (-0.87 - 0.98)	-0.36 (-1.49 - 0.78)
**In-person visit rate # period**			
100% - post	1.06 (-0.64 - 2.75)	1.12 (-0.36 - 2.60)	-0.56 (-2.16 - 1.03)
**Practice size category (ref = solo)[e]**			
Small (2-5)	-1.66 (-3.16 - -0.15)	-0.48 (-1.52 - 0.56)	-1.13 (-2.03 - -0.24)
Medium (6-20)	0.08 (-2.02 - 2.19)	1.02 (-0.42 - 2.46)	-0.92 (-2.02 - 0.17)
Large (21+)	1.76 (-1.96 - 5.48)	0.88 (-0.79 - 2.56)	0.99 (-1.23 - 3.21)
TIN percent with broadband	-0.16 (-0.23 - -0.09)	-0.09 (-0.13 - -0.05)	-0.07 (-0.10 - -0.03)
TIN rural	0.07 (-2.23 - 2.38)	1.27 (0.08 - 2.46)	-1.11 (-2.45 - 0.24)
Female	9.85 (8.62 - 11.08)	9.28 (7.80 - 10.77)	0.73 (0.34 - 1.12)
Age	0.09 (0.06 - 0.12)	0.11 (0.09 - 0.13)	-0.03 (-0.05 - -0.01)
**Comorbidities[d]**			
Cancer	5.45 (4.30 - 6.59)	6.58 (5.37 - 7.79)	-0.47 (-1.16 - 0.22)
Diabetes	25.28 (16.01 - 34.55)	4.39 (3.52 - 5.26)	23.82 (12.71 - 34.94)
Immune compromise	8.99 (6.69 - 11.29)	7.79 (6.08 - 9.51)	1.17 (-0.05 - 2.38)
CHF	15.82 (13.91 - 17.73)	7.09 (5.62 - 8.55)	8.56 (7.40 - 9.72)
COPD	27.45 (25.35 - 29.55)	9.66 (8.32 - 11.00)	17.82 (15.99 - 19.66)
Renal insufficiency	56.93 (52.59 - 61.27)	60.74 (56.27 - 65.21)	8.01 (6.82 - 9.21)

Source: Analysis of Blue Cross Blue Shield of Michigan claims, 2019-2020
[a]ACSC: Emergency department visits or hospitalizations for ambulatory care-sensitive conditions
[b]Interaction between any TH use (yes vs no) and the time period (pre vs post)
[c]Proportion of care provided in-person in March-Aug 2020 compared to monthly visits January 2019-February 2020
[d]Comorbidities identified from Hierarchical Condition Categories
[e]Practice size determined by number of National Provider Identifier (NPI) numbers associated with the same Tax Identification Number (TIN)

© 2022 Li KY et al. *JAMA Network Open.*

eTable 3 Average marginal effects of practice telehealth use (continuous) and practice- and patient-level characteristics on estimated ACSC visits per 1000 patients per year

Attribute	Any ACSC[b] AME (95% CI)	Acute ACSCs AME (95% CI)	Chronic ACSCs AME (95% CI)
**Telehealth use rate # period**[b]			
100% - post	1.02 (-1.73 - 3.77)	0.42 (-1.73 - 2.57)	0.38 (-1.3 - 2.06)
**Telehealth use rate**	6.61 (2.56 - 10.66)	4.38 (1.8 - 6.95)	2.45 (0.3 - 4.6)
**Period (ref=Jun-Sep 2019)**			
Post (Jun-Sep 2020)	-8.4 (-9.08 - -7.73)	-5.32 (-5.75 - -4.88)	-3.39 (-3.95 - -2.83)
**In-person visit rate**[c]	0.25 (-0.95 - 1.45)	0.17 (-0.7 - 1.04)	-0.03 (-0.87 - 0.8)
**In-person visit rate # period**			
100% - post	1.21 (-0.33 - 2.74)	1.18 (-0.16 - 2.53)	-0.24 (-1.24 - 0.75)
**Practice size category (ref = solo)**[d]			
Small (2-5)	-1.63 (-3.12 - -0.15)	-0.44 (-1.47 - 0.59)	-1.13 (-2.01 - -0.25)
Medium (6-20)	0.22 (-1.86 - 2.3)	1.16 (-0.27 - 2.6)	-0.92 (-1.98 - 0.14)
Large (21+)	1.87 (-1.74 - 5.49)	1.02 (-0.6 - 2.64)	1 (-1.16 - 3.17)
TIN percent with broadband	-0.15 (-0.23 - -0.08)	-0.09 (-0.13 - -0.05)	-0.07 (-0.1 - -0.03)
TIN rural	0.51 (-1.68 - 2.69)	1.5 (0.43 - 2.56)	-0.95 (-2.24 - 0.35)
Female	9.83 (8.61 - 11.05)	9.26 (7.79 - 10.74)	0.73 (0.34 - 1.12)
Age	0.09 (0.06 - 0.11)	0.11 (0.09 - 0.12)	-0.04 (-0.06 - -0.02)
**Comorbidities**[e]			
Cancer	5.55 (4.37 - 6.73)	6.68 (5.45 - 7.91)	-0.45 (-1.14 - 0.24)
Diabetes	25.15 (15.94 - 34.36)	4.31 (3.44 - 5.17)	23.74 (12.71 - 34.78)
Immune compromise	8.89 (6.57 - 11.21)	7.68 (5.96 - 9.41)	1.18 (-0.05 - 2.42)
CHF	15.82 (13.91 - 17.72)	7.11 (5.65 - 8.57)	8.54 (7.38 - 9.7)
COPD	27.4 (25.27 - 29.54)	9.63 (8.28 - 10.98)	17.8 (15.93 - 19.67)
Renal insufficiency	56.64 (52.22 - 61.05)	60.42 (55.93 - 64.91)	7.97 (6.77 - 9.17)

Source: Analysis of Blue Cross Blue Shield of Michigan claims, 2019-2020
[a]ACSC: Emergency department visits or hospitalizations for ambulatory care-sensitive conditions
[b]Interaction between the rate of practice telehealth use (continuous) and the time period (pre vs post)
[c]Proportion of care provided in-person in March-Aug 2020 compared to monthly visits January 2019-February 2020
[d]Practice size determined by number of National Provider Identifier (NPI) numbers associated with the same Tax Identification Number (TIN)
[e]Comorbidities identified from Hierarchical Condition Categories

© 2022 Li KY et al. *JAMA Network Open.*
